# Determinants and Prevalence of Depression Among Dietary Supplement Users in Saudi Arabia: A Cross-Sectional Study

**DOI:** 10.7759/cureus.56736

**Published:** 2024-03-22

**Authors:** Aeshah Alharbi, Ahmad Aldosary, Farah Alsuwailem, Lama Alhumaidan, Norah Alharbi

**Affiliations:** 1 College of Medicine, Unaizah College of Medicine and Medical Sciences, Qassim University, Unaizah, SAU; 2 Department of Family and Community Medicine, College of Medicine, Qassim University, Unaizah, SAU

**Keywords:** cross-sectional study, depression, saudi arabia, depressive symptoms, dietary supplements

## Abstract

Background: Dietary supplements (DSs) are used by a large number of people globally. It is widely believed that DSs can improve health, prevent diseases, and replenish vitamin and micronutrient deficiencies. We aimed to determine the prevalence of and factors associated with DS use among the Saudi population and the association between DS consumption and depressive symptoms.

Research methodology: This observational cross-sectional study was conducted in 2022. The questionnaire was distributed through an online mode among adult Saudis (18 years or older) living in the Kingdom of Saudi Arabia. The survey included four parts: socio-demographic characteristics, participants’ health, lifestyle, and activity, vitamin and mineral supplement use, and a patient health questionnaire (PHQ-9) as a valid and reliable diagnostic tool for depression.

Result: Of the 1309 respondents, 1173 were enrolled. The mean age of the participants was 26.3 ± 8.8 (range, 18-24) years, and approximately 14.7% exhibited psychological anxiety while 8.4% experienced psychological depression. The prevalence of DS use among Saudis was found to be relatively high (52.2%). The most commonly used DS was vitamin D (43%). DSs improved depressive symptoms in 49.4% of the participants.

Conclusion: The prevalence of DS use is high among the Saudi population, and vitamin D is the most common DS. The use of multivitamins and minerals, especially iron, is associated with improved depressive symptoms; however, further studies are needed to understand the relationship between DS use and depressive symptoms.

## Introduction

Billions of people worldwide take dietary supplements (DSs), such as multivitamin-multimineral (MVMM) preparations to improve their health, prevent diseases, and replenish vitamin and mineral deficiencies [1]. A DS must contain one or more of the following: vitamins, minerals, amino acids, herbs, food components, metabolites, and extracts that can provide nutritional or physiological benefits to the body [2]. MVMM consumption is common in Saudi Arabia, and people consider that these preparations can help them stay healthy [3]. A previous study showed that 47% of the general Saudi population consumes MVMM supplements [4].

Depression is a leading cause of disability worldwide and the fourth leading cause of early death [5]. It has been suggested that a poor diet is associated with poor mental health, including depression [6]. Although there are several effective therapies for depression, researching alternate therapies or preventative measures is necessary [7]. Dietary therapies and vitamin supplementation, particularly vitamin B supplementation, are among the most popular complementary and alternative medical interventions for depression [8]. Clinical studies have shown that patients with a low folate status have a higher risk of developing depression and worsening depressive symptoms and exhibit a lower response to antidepressants [9]. Besides depression, several other factors are also associated with DS consumption. A cross-sectional study showed that 71% of individuals who consume DS are females, older adults, and highly educated [10]. Regular exercising is shown to be associated with DS consumption [11] and DS usage is more prevalent among athletes than non-athletes. Moreover, the use of DSs is higher among non-smokers than in smokers [12].

A previous study showed a link between depressive symptoms and vitamin inadequacies in Japanese individuals [13]. A study conducted in Saudi Arabia during the coronavirus disease 2019 (COVID-19) pandemic showed that more than 25% of the participants had depression, and the majority of them were not using any DS [14]. Another study from Saudi Arabia showed that MVMM use is significantly correlated with the sex, marital status, educational levels, regular exercise, smoking status special diet consumption, and fruit and vegetable consumption [4]. We aimed to investigate the correlation between DS intake and relevant factors, including depressive symptoms. Our study provides an understanding of DS use among adult Saudis and the factors influencing the use of these supplements.

## Materials and methods

This observational cross-sectional study was conducted from July 21, 2022, to September 2, 2022, using an online survey. Out of 1309, a total of 1173 of the participants were included in the current study. The study participants were adult Saudis aged ≥18 years living in the Kingdom of Saudi Arabia (KSA). The study was conducted in accordance with the guidelines of the Declaration of Helsinki and approved by the Regional Ethics Committee of the Qassim Region, KSA (21-18-11). A self-administered structured survey instrument was developed by our research team based on published work in this field. The survey included four parts: socio-demographic characteristics (age, sex, residence, marital status, level of education, occupation), and we also collected data on participants’ self-reported height and weight to determine their body mass index (BMI) following the WHO classification for adults. The healthy adult BMI range is 18.5-24.9 kg/m^2^, with those below the range being considered underweight. Those whose BMI is between 25.0 and 29.9 kg/m^2^ are overweight, and those whose BMI is above 30 kg/m^2^ are obese. Participants’ health, lifestyle, and activity status (health status, diet, eating healthy food, exercise, and chronic conditions), vitamin and mineral supplement use (type of DS, frequency of use, source of these products, and reason for using DSs), and a patient health questionnaire (PHQ-9) were used as a valid and reliable diagnostic tool for depression. In the first three sections, we used a modified questionnaire adapted from the study by Alwalan et al. [4]. The validity of the survey instrument was ensured through a pilot study on ten randomly selected Saudis. The questionnaire was then translated into Arabic. Data were collected after obtaining ethical approval from the research ethics committee. The questionnaire was distributed online among participants selected using the snowball sampling method. Participation was voluntary, and electronic informed consent was obtained before data collection. Once consent was provided, they completed the online questionnaire. The participants were assured confidentiality and were free to withdraw from the study at any time. The data were collected, organized, tabulated, and analyzed using IBM SPSS Statistics for Windows, Version 23 (Released 2015; IBM Corp., Armonk, New York, United States). Categorical data were expressed as numbers and percentages. The chi-squared (χ2) test was used to assess the relationship between two or more qualitative variables. Quantitative variables were analyzed using the Mann-Whitney test. Quantitative data were expressed as a p-value < 0.05, indicating statistical significance.

## Results

Socio-demographic characteristics of study participants

Out of 1309, a total of 1173 participants were included in the current study. The mean age of the participants was found to be 26.3 ± 8.8 (range of 18 - 24) years old. The mean body mass index was found to be 24.6 ± 5.9 (range of 12.4 - 75.6). About 805 (68.6%) of the participants were females and the rest 368 (31.4%) were males. About 586 (50%) were with a healthy weight (BMI of 18.5-24.9), 287 (24.5%) were considered to be overweight (BMI of 25 - 29.9), 175 (14.9%) were obese (BMI of > 30), and 125 (10.7%) of them were underweight. Marital status of 829 participants (70.7%) was found to be single, 309 (26.3%) were married, 23 (2%) were divorced, and 12 (1%) were widowed. Six hundred and six participants (51.7%) were students, 232 (19.8%) were unemployed, 165 (14.1%) were private sector employees, 150 (12.8%) were governmental employees, and 20 (1.7%) were retired. Regarding the educational level, most of the participants 682 (58.1%) had a bachelor's degree, 329 (28%) had a secondary educational level, 94 (8%) had a diploma, 48 (4.1%) had postgraduate, 10 (0.9%) had an intermediate level, 8 (0.7%) had a primary educational level, and 2 (0.2%) were illiterate. Monthly total household income (SAR) was found to be less than 5,000 SR in 825 (70.3%) of the participants. 5,000- 9,999 SR in 179 (15.3%) of the participants, 10,000-15,000 in 105 (9%) of them, and more than 15,000 in 64 (5.5%) of the participants (Table 1).

**Table 1:** Socio-demographic characteristics of the study participants (n = 1,173). SD: standard deviation; BMI: body mass index; SAR: Saudi Arabian Riyal

Variable		Mean ± SD	Range
Age (years)		26.3 ± 8.8	18–64
BMI (kg/m^2^)		24.6 ± 5.9	12.4–75.6
		Frequency	Percentage
Sex	Male	368	31.4%
	Female	805	68.6%
BMI (kg/m^2^)	Underweight (<18.5)	125	10.7%
	Healthy (18.5–24.9)	586	50%
	Overweight (25–29.9)	287	24.5%
	Obese (>30)	175	14.9%
Marital status	Single	829	70.7%
	Married	309	26.3%
	Divorced	23	2%
	Widowed	12	1%
Employment status	Student	606	51.7%
	Government employee	150	12.8%
	Private sector employee	165	14.1%
	Unemployed	232	19.8%
	Retired	20	1.7%
Educational level	Illiterate	2	0.2%
	Primary	8	0.7%
	Intermediate	10	0.9%
	Secondary	329	28%
	Diploma	94	8%
	Bachelor’s degree	682	58.1%
	Postgraduate	48	4.1%
Monthly total household income (SAR)	Less than 5,000	825	70.3%
	5,000–9,999	179	15.3%
	10,000–15,000	105	9%
	More than 15,000	64	5.5%

Lifestyle and activity

Of the total participants, 632 (53.9%) rated their health as good, 479 (40.8%) rated their health as excellent, and 62 (5.3%) rated their health as poor. Furthermore, 260 (22.2%) participants followed a special diet while the remaining 913 did not follow any special diet. Moreover, 650 (55.4%) participants usually ate fruits and vegetables, and among them, 231 (35.5%) usually had a daily intake of vegetables, 151 (23.2%) ate vegetables three times per week, 122 (18.8%) twice a week, 81 (12.5%) once every two weeks, and 65 (10%) once every week. Regarding exercise, 463 (39.5%) participants exercised sometimes, 342 (29.2%) rarely exercised, 168 (14.3%) always exercised, and 200 (17.1%) never exercised. With respect to the type of exercise, walking was preferred by 767 (78.8%) participants, mixed sports by 278 (28.6%) participants, jogging by 166 (17.1%) participants, running by 130 (13.4%) participants, swimming by 89 (9.1%) participants, and cycling by 62 (6.4%) participants. Additionally, 129 (11%) participants were smokers, 41 (3.5%) were ex-smokers, and 1003 (85.5%) were non-smokers (Table 2).

**Table 2:** Lifestyle and activity.

Questions	Response	Frequency	Percentage
In general, how would you rate your health?	Excellent	479	40.8%
	Good	632	53.9%
	Poor	62	5.3%
Do you currently follow a special diet?	Yes	260	22.2%
	No	913	77.8%
Do you usually eat fruits and vegetables?	Yes	650	55.4%
	No	523	44.6%
If your answer is yes, how often? (n = 650)	Once every week	65	10%
	Once in two weeks	81	12.5%
	Twice a week	122	18.8%
	Three times a week	151	23.2%
	Daily	231	35.5%
Do you exercise regularly? And how often do you exercise?	No	200	17.1%
	Rarely	342	29.2%
	Sometimes	463	39.5%
	Always	168	14.3%
If yes, what exercises do you perform? (n = 973)	Walking	767	78.8%
	Jogging	166	17.1%
	Running	130	13.4%
	Cycling	62	6.4%
	Swimming	89	9.1%
	Weightlifting	158	16.2%
	Mixed sports	278	28.6%
Do you smoke?	Yes	129	11%
	No	1003	85.5%
	Ex-smoker	41	3.5%

**Figure 1:**
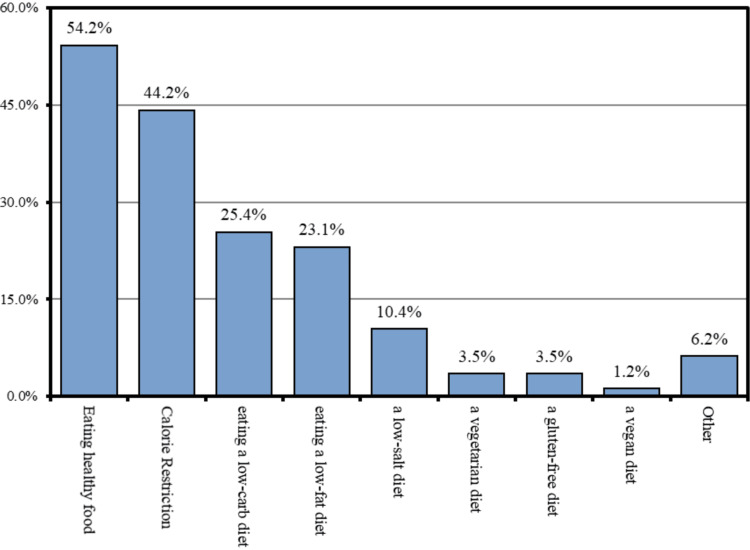
Special diets consumed by the study participants.

Of the 260 participants who consumed a special diet, 141 (54.2%) preferred eating healthy foods, 115 (44.2%) preferred calorie restriction, 66 (25.4%) preferred a low-carb diet, 60 (23.1%) preferred a sated low-fat diet, 27 (10.4%) preferred a low-salt diet, 9 (3.5%) preferred a vegetarian gluten-free diet, 3 (1.2%) preferred a vegan diet, and 16 (6.2%) preferred other diets (Figure 1).

**Figure 2:**
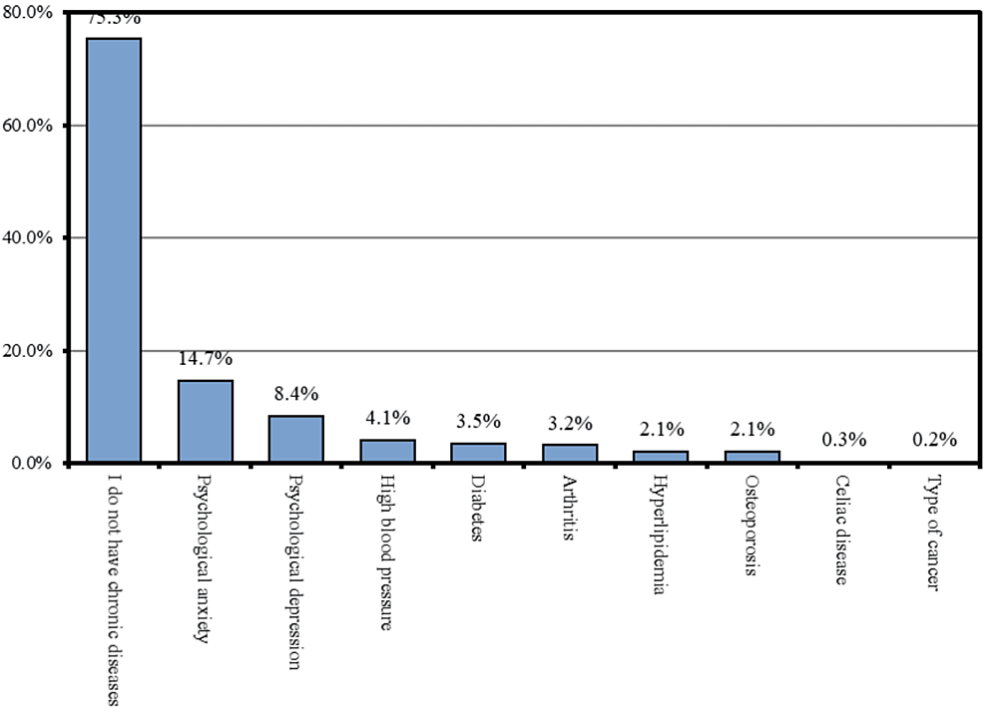
The prevalence of chronic diseases among study participants.

With respect to disease status, 883 (75.3%) participants had no chronic diseases, 172 (14.7%) reported psychological anxiety, 99 (8.4%) reported psychological depression, 48 (4.1%) had hypertension, 41 (3.5%) had diabetes, 37 (3.2%) had arthritis, 25 (2.1%) had hyperlipidemia, 25 (2.1%) had osteoporosis, 4 (0.3%) reported celiac disease, and 2 (0.2%) had cancer (Figure 2).

Prevalence of DS use among the Saudi population

**Figure 3:**
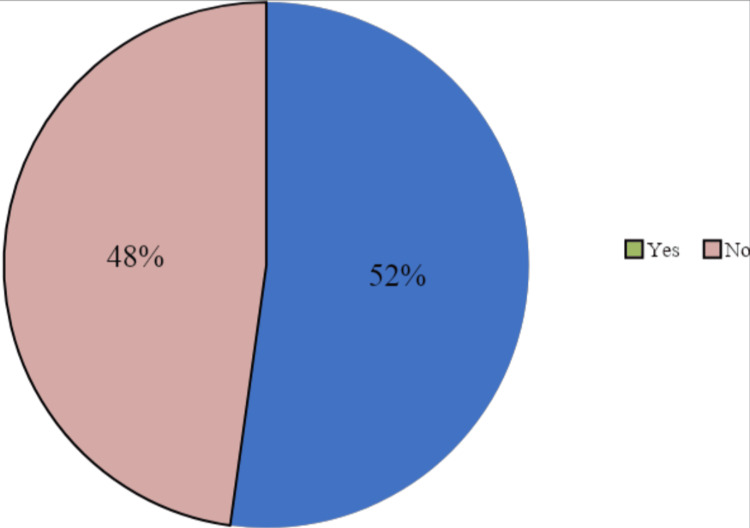
Dietary supplement use among the Saudi population.

The prevalence of DS use among the Saudi population was found to be relatively high, as 612 (52.2%) participants used DSs regularly for more than one month (Figure 3).

**Figure 4:**
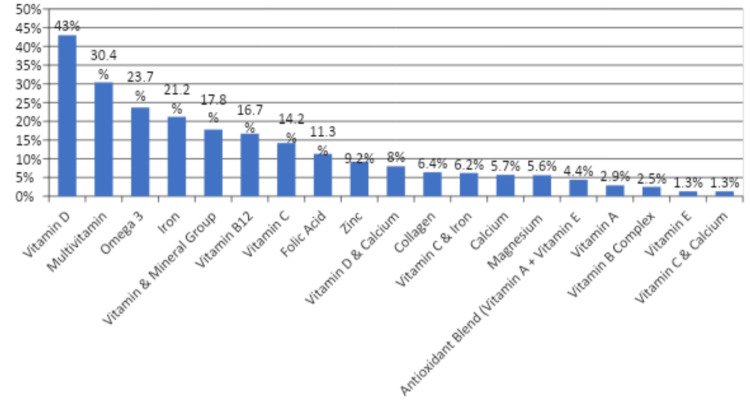
Types of dietary supplements used by the study participants.

Regarding the type of DS, 263 (43%) participants used vitamin D, 186 (30.4%) used multivitamins, 145 (23.7%) used omega-3 supplements, 130 (21.2%) used iron supplements, 109 (17.8%) were taking vitamins and minerals, 102 (16.7%) used vitamin B12, 87 (14.2%) used vitamin C, 56 (9.2%) used zinc supplements, 49 (8%) used vitamin D and calcium, 39 (6.4%) used collagen, 38 (6.2%) used vitamin C and iron, 35 (5.7%) used calcium, 34 (5.6%) used magnesium, 27 (4.4%) used antioxidants, 18 (2.9%) used vitamin A, 15 (2.5%) used vitamin B complex, 8 (1.3%) used vitamin E, and 8 (1.3%) reported using vitamin C and calcium (Figure 4).

Among the study population, 393 (64.2%) used DSs on a daily basis, 177 (28.9%) used DSs every week, 35 (5.7%) used them every month, and 7 (1.1%) were not sure. Moreover, 297 (48.5%) participants obtained these products from hospitals with a prescription, 124 (20.3%) obtained them from a pharmacy without a prescription, 141 (23%) ordered these products online, 21 (3.4%) got these products from complementary health stores, 9 (1.5%) from trainers in the gym, 8 (1.3) from social media and friends, and 4 (0.7%) from other sources. Interestingly, 532 (45.4%) participants believed that these products might help in maintaining good health, 464 (39.6%) believed that these supplements helped a lot, 109 (9.3%) did not think these mattered, and 68 (5.8%) believed that these did not do any harm. The general effects of using one of these products were improving and maintaining the general form of health as reported by 750 (63.9%) participants, preventing the occurrence of diseases related to the heart and blood vessels as mentioned by 43 (3.7%) participants, and preventing the occurrence of some cancers as reported by 17 (1.4%) participants, and 363 (30.9%) participants were not sure. Finally, 580 (49.4%) participants believed that DSs improved the symptoms of depression, and 228 (17.4%) did not think that DSs improved depressive symptoms, whereas 365 (31.1%) were not sure (Table 3).

**Table 3:** Pattern of dietary supplement use among the Saudi population.

Variable Question	Categories Response	Frequency	Percentage
How many times do you use these dietary supplements? (n = 612)	On a daily basis	393	64.2%
	Every week	177	28.9%
	Every month	35	5.7%
	I am not sure	7	1.1%
From where did you get these products? (n = 612)	Hospital withas a prescription	297	48.5%
	Pharmacy without a prescription	124	20.3%
	Complementary health stores	21	3.4%
	From the internet	141	23%
	Social media	8	1.3%
	A friend of minee	8	1.3%
	Trainers in the gym	9	1.5%
	Others	4	0.7%
What do you think is the importance of these products in maintaining health?	I don't think it matters	109	9.3%
	It doesn't do me any harm	68	5.8%
	It might help	532	45.4%
	It helps a lot	464	39.6%
What are the general health effects of using one of these products?	Maintaining and improving the general form of health	750	63.9%
	Preventing the occurrence of diseases related to the heart and blood vessels	43	3.7%
	Preventing the occurrence of some cancers	17	1.4%
	I don't know.	363	30.9%
Do you think that dietary supplements may help in improving the symptoms of depression?	Yes	580	49.4%
	No	228	19.4%
	I don’t know	365	31.1%

**Figure 5:**
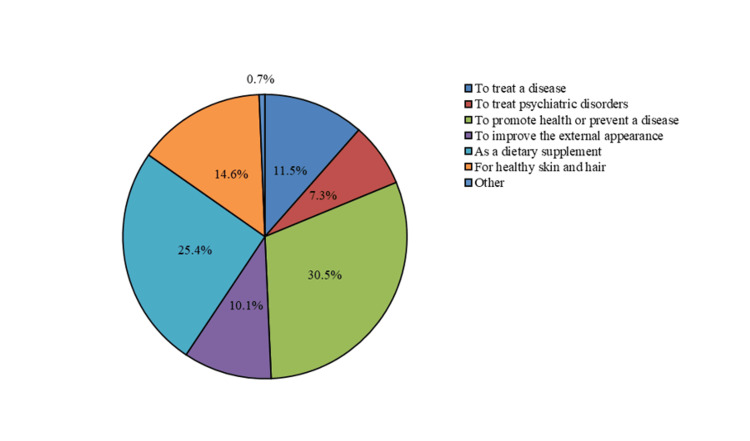
Purpose of using dietary supplements.

Regarding the purpose of using DSs, 431 (30.5%) participants used them to promote health or prevent a disease, 358 (25.4%) as a DS, 206 (14.6%) for healthy skin and hair, 162 (11.5%) to treat diseases, 142 (10.1%) to improve external appearance, 103 (7.3%) to treat psychiatric disorders, and 9 (0.7%) for other purposes (Figure 5).

Sex was found to be significantly associated with DS use among the Saudi Arabian population (p < 0.001), with females tending to use these products more than males. BMI was found to be significantly associated with the use of DSs (p = 0.002), with underweight and healthy participants using supplements more frequently than others. Marital status and the use of DSs were found to be significantly associated (p = 0.024), as divorced and widowed participants tended to use DSs more frequently. The educational level and the use of DSs were found to be significantly associated (p = 0.045) with the postgraduate group using DSs more frequently than others. Health status was also found to be significantly associated with DS use (p = 0.009), as participants who thought that their health was bad tended to use DSs more frequently. Special diet consumption and using DSs were found to be significantly associated (p < 0.001). Participants following special diets tended to use vitamin supplements more frequently than others. A statistically significant association was found between smoking status and the use of DSs (p = 0.017), as non-smokers tended to use DSs more frequently. Employment status, monthly income, and regular exercising were not significantly associated with DS use (p = 0.380, 0.138, and 0.162, respectively) (Table 4).

**Table 4:** Factors associated with dietary supplement use among the Saudi population.

Variable		Dietary supplement use		p-value [A1]
		Yes	No	
		Mean (SD)		
Age (years)		26.5 (8.8)	26.1 (8.9)	0.493
		N (%)		
Gender (Sex)	Male	139 (37.8)	229 (62.2)	< 0.001
	Female	473 (58.8)	332 (41.2)	
Body mass index (BMI)	Underweight	70 (56)	55 (44)	0.002
	Healthy weight	332 (56.7)	254 (43.3)	
	Overweight	124 (43.2)	163 (56.8)	
	Obese	89 (49.1)	86 (49.1)	
Marital status	Single	409 (49.3)	420 (50.7)	0.024
	Married	181 (58.6)	128 (41.4)	
	Divorced	14 (60.9)	9 (39.1)	
	Widowed	8 (66.7)	4 (33.3)	
Employment status	Student	305 (50.3%)	301 (49.7)	0.380
	Government employee	78 (52)	72 (48)	
	Private sector employee	123 (53)	109 (47)	
	Unemployed	9 (45)	11 (55)	
	Retired	97 (58.8)	68 (41.2)	
Educational level	Illiterate	1 (50)	1 (50)	0.045
	Primary	5 (62.5)	3 (37.5)	
	Intermediate	7 (70)	3 (30)	
	Secondary	165 (50.2)	164 (49.8)	
	Diploma	37 (39.4)	57 (60.6)	
	Bachelor's degree	365 (53.5)	317 (46.5)	
	Postgraduate studies	32 (66.7)	16 (33.3)	
Monthly total household income (SAAR)	Less than 5,000	414 (50.2)	411 (49.8)	0.138
	5,000 –- 9,999	97 (54.2)	82 (45.8)	
	10,000 –- 15,000	63 (60)	42 (40)	
	More than 15,000	38 (59.4)	26 (40.6)	
In general, how would you rate your health?	Excellent	232 (48.4%)	247 (51.6)	0.009
	Good	345 (54.6)	287 (45.4)	
	Bad/Poor	35 (56.5)	27 (43.5)	
Do you currently follow a special diet?	Yes	172 (66.2)	88 (33.8)	< 0.001
	No	440 (48.2)	473 (51.8)	
Do you usually eat fruits and vegetables?	Yes	356 (54.8)	294 (45.2)	0.047
	No	256 (48.9)	267 (51.1)	
Do you exercise regularly? And how often do you exercise?	No	96 (48)	104 (52)	0.162
	Rarely	169 (49.4)	173 (50.6)	
	Sometimes	250 (54)	213 (46)	
	Always	97 (57.7)	71 (42.3)	
Do you smoke?	Yes	52 (40.3)	77 (59.7)	0.017
	No	538 (53.6)	465 (46.4)	
	Ex-smoker	22 (53.7)	19 (46.3)	

PHQ-9 for depression

**Figure 6:**
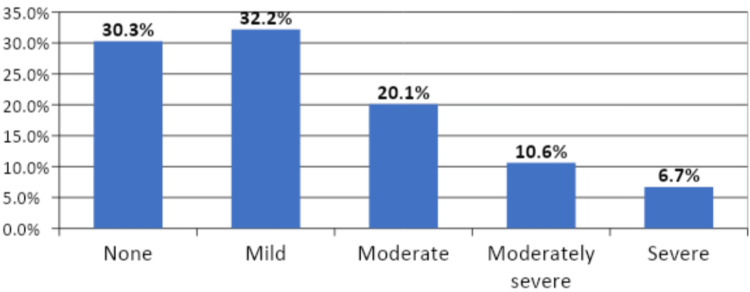
Depression severity among the study participants.

The average PHQ-9 score of the participants was found to be 8.54 ± 6.16 (range, 0-27). Concerning severity, 378 (32.2%) participants had mild depression, 236 (20.1%) reported moderate depression, 124 (10.6%) exhibited moderately severe depression, 79 (6.7%) had severe depression, and 356 (30.3%) participants did not have depression (Figure 6).

Association between DS consumption and depressive symptoms

A significant correlation was observed between multivitamin use and PHQ-9 scores (p = 0.024). Participants using multivitamins had a lower PHQ-9 score and fewer depressive symptoms than others. Vitamin C use was correlated with PHQ-9 scores (p ˂ 0.001), as participants who were not taking vitamin C tended to have lower PHQ-9 scores and lesser depressive symptoms than did those who were taking it. The use of vitamin B12, vitamin A, vitamin D, and zinc was found to be significantly associated with the PHQ-9 score (p = 0.002, 0.040, 0.013, and 0.028, respectively). Participants who did not use these supplements tended to have fewer depressive symptoms than did those who did. The intake of vitamins and minerals and iron supplements was significantly associated with the PHQ-9 score (p = 0.001 and 0.025, respectively); participants taking these products tended to have fewer depressive symptoms than others. Vitamin E, folic acid, calcium, omega-3 supplement, and collagen use showed no significant association with PHQ-9 scores (p = 0.169, 0.710, 0.299, 0.130, and 0.089, respectively) (Table 5).

**Table 5:** Association between dietary supplement consumption and depressive symptoms. *p-values were calculated using the Mann–Whitney test.

Variable		N	PHQ-9 Score		p-value
			Mean	SD	
Using dietary supplements	No	561	8.35	6.16	0.286
	Yes	612	8.72	6.16	
Multivitamin	No	426	8.97	5.94	0.024
	Yes	186	8.13	6.61	
Vitamin C	No	525	8.35	6.16	< 0.001
	Yes	87	10.94	5.71	
Vitamin B12	No	510	8.36	6.03	0.002
	Yes	102	10.47	6.50	
Vitamin A	No	594	8.63	6.15	0.040
	Yes	18	11.44	5.93	
Vitamin E	No	604	8.67	6.14	0.169
	Yes	8	12.00	7.13	
Vitamin B cComplex	No	597	8.65	6.14	0.094
	Yes	15	11.47	6.66	
Folic aAcid	No	543	8.70	6.20	0.710
	Yes	69	8.87	5.84	
Antioxidant bBlend (vVitamin A + vVitamin E + vVitamin C)]	No	585	8.75	6.17	0.442
	Yes	27	7.93	6.02	
Vitamin & mineral group	No	503	9.10	6.20	0.001
	Yes	109	6.94	5.68	
Vitamin C & iIron	No	574	8.58	6.07	0.083
	Yes	38	10.76	7.21	
Vitamin D	No	349	8.16	5.95	0.013
	Yes	263	9.45	6.36	
Vitamin D & cCalcium	No	563	8.74	6.17	0.786
	Yes	49	8.43	6.03	
Vitamin C & cCalcium	No	604	8.70	6.16	0.574
	Yes	8	9.88	6.36	
Calcium	No	577	8.77	6.16	0.299
	Yes	35	7.74	6.12	
Iron	No	482	8.43	6.10	0.025
	Yes	130	9.77	6.29	
Zinc	No	556	8.51	6.02	0.028
	Yes	56	10.77	7.13	
Magnesium	No	578	8.66	6.10	0.533
	Yes	34	9.59	7.08	
Omega- 3	No	467	8.88	6.10	0.130
	Yes	145	8.19	6.32	
Collagen	No	573	8.83	6.20	0.081
	Yes	39	7.08	5.33	

## Discussion

A balanced intake of various types of nutrients is key to a healthy life. Importantly, body requirements of proteins, carbohydrates, and fats should be fulfilled. Vitamins and minerals are essential micronutrients required by the body.

We aimed to determine the prevalence of DS use, associated factors, types of DSs used, and depressive symptoms among the Saudi population. MVMM consumption is common in Saudi Arabia and people consider that these preparations might help them to stay healthy. In our study, the prevalence of DS use was found to be 52.2%, and these participants used DSs regularly. Similar to our study, a previous cross-sectional study conducted in Riyadh, KSA, showed that nearly half of the general population consumes MVMM supplements [3], whereas the prevalence of nutritional supplement use among the Chinese population was 0.71% [15]. Thus, there is a considerable difference in DS use between Chinese and Saudi populations. In the United States, the use of DSs has consistently increased over the past 40 years, with 49% of the general population and 70% of individuals over the age of 70 years reportedly consuming DSs [16]. Several factors could affect the use of DSs. We observed that DS consumption was significantly higher in females than in males. Similar findings have been reported in previous studies [3,4,17]. In general, women are more health conscious than men. Women use DSs during pregnancy and for purely cosmetic purposes to maintain their hair and skin health. Furthermore, women frequently use supplemental calcium, vitamin D, and iron for maintaining bone health, preventing the onset of osteoporosis during aging, and reducing the risk of developing anemia [18,19]. We found that DS intake was higher in individuals with a high level of education than others, which is similar to the results of the study by Alhashem et al. showing that approximately 85.5% of individuals with higher education prefer consuming DSs [17]. The National Health and Nutrition Examination Survey (NHANES) reported that 61% of individuals with more than a high-school education grade and 37% of those with less than high-school educational level used DSs [16]. Our study showed that the majority of DS users were non-smokers, which is in accordance with the results of a previous study [3]. Interestingly, we observed that the use of DSs was more among divorced and widowed participants than others, which is contradictory to the findings of a previous study carried out in Qatar reporting no significant association between marital status and DS use [20]. However, another cross-sectional study on female college students showed a higher use of supplements among single female students than among married females [21]. Our results showed that underweight and healthy participants and those who thought that their health was poor used supplements more frequently than others. It is widely believed that some DSs can help in weight gain, which could explain the increased use of DSs in these groups. Our study showed that DS use was not significantly associated with employment status, monthly income, and regular exercise. However, previous studies showed that regular exercise is positively associated with DS consumption. It has also been reported that DS usage is more prevalent among athletes than non-athletes [11]. Nearly half of our study participants believed that DSs could improve depressive symptoms, 17.4% of the participants believed that DS did not improve depression symptoms, and nearly one-third had no idea about this relationship. Similar results were reported by Gariballa et al. who showed that the use of DSs improves depressive symptoms [22]. PHQ-9 scores reflect the severity of depressive symptoms. We observed that participants using multivitamins had lower PHQ-9 scores and fewer depressive symptoms than others. Nguyen et al. also reported that multivitamin use is associated with improved depressive symptoms [23]. Furthermore, participants taking vitamins & minerals and iron supplements exhibited fewer depressive symptoms than those with no intake. The use of vitamin E, folic acid, omega-3 supplements, collagen, and calcium showed no significant association with the PHQ-9 score. Okronipa et al. also reported that the uptake of folic acid, calcium, and essential fatty acids is not associated with depressive symptoms [24].

The main limitation of this study is its cross-sectional design, which makes it difficult to determine the significance of the association between DS use and depressive symptoms. Another limitation was the snowball sampling method as it posed a risk of several biases.

## Conclusions

The prevalence of DS use is high among the Saudi population, and vitamin D is the most common DS. The prevalence of depression is approximately 8.4% among Saudis. The use of multivitamins and minerals, especially iron, is associated with improved depressive symptoms; however, further studies are required to confirm this relationship.
